# Initial investigation using statistical process control for quality control of accelerator beam steering

**DOI:** 10.1186/1748-717X-6-180

**Published:** 2011-12-28

**Authors:** Charles M Able, Carnell J Hampton, Alan H Baydush, Michael T Munley

**Affiliations:** 1 Department of Radiation Oncology, Wake Forest School of Medicine, Medical Center Boulevard, Winston-Salem, NC 27157, USA

**Keywords:** Quality control, quality assurance, statistical process control, radiation therapy

## Abstract

**Background:**

This study seeks to increase clinical operational efficiency and accelerator beam consistency by retrospectively investigating the application of statistical process control (SPC) to linear accelerator beam steering parameters to determine the utility of such a methodology in detecting changes prior to equipment failure (interlocks actuated).

**Methods:**

Steering coil currents (SCC) for the transverse and radial planes are set such that a reproducibly useful photon or electron beam is available. SCC are sampled and stored in the control console computer each day during the morning warm-up. The transverse and radial - positioning and angle SCC for photon beam energies were evaluated using average and range (Xbar-R) process control charts (PCC). The weekly average and range values (subgroup n = 5) for each steering coil were used to develop the PCC. SCC from September 2009 (annual calibration) until two weeks following a beam steering failure in June 2010 were evaluated. PCC limits were calculated using the first twenty subgroups. Appropriate action limits were developed using conventional SPC guidelines.

**Results:**

PCC high-alarm action limit was set at 6 standard deviations from the mean. A value exceeding this limit would require beam scanning and evaluation by the physicist and engineer. Two low alarms were used to indicate negative trends. Alarms received following establishment of limits (week 20) are indicative of a non-random cause for deviation (Xbar chart) and/or an uncontrolled process (R chart). Transverse angle SCC for 6 MV and 15 MV indicated a high-alarm 90 and 108 days prior to equipment failure respectively. A downward trend in this parameter continued, with high-alarm, until failure. Transverse position and radial angle SCC for 6 and 15 MV indicated low-alarms starting as early as 124 and 116 days prior to failure, respectively.

**Conclusion:**

Radiotherapy clinical efficiency and accelerator beam consistency may be improved by instituting SPC methods to monitor the beam steering process and detect abnormal changes prior to equipment failure.

**PACS numbers: **87.55n, 87.55qr, 87.56bd

## I. Background

**Figure 1:**
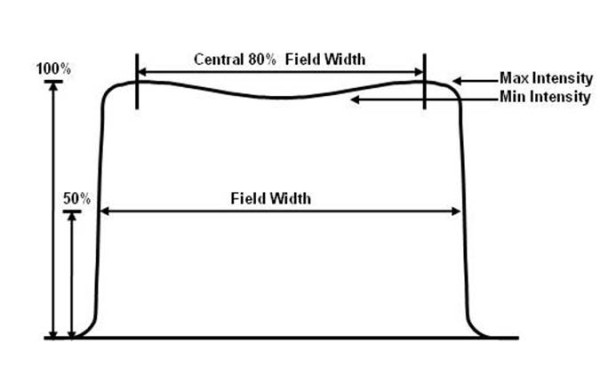
**Idealized radiation beam intensity profile of a typical accelerator produced photon beam**. Beam flatness is the maximum plus and minus variation from the mean beam intensity within the central 80% area of full width half maximum intensity and linear symmetry is defined as the maximum variation of points symmetric to the central axis.

**Figure 2:**
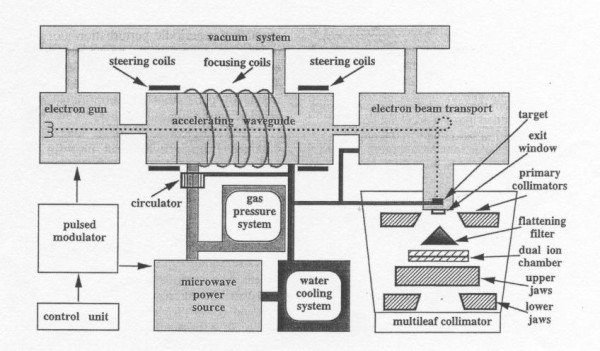
**Schematic diagram of a typical medical linear accelerator**. (reproduced from Van Dyk, J. The modern technology of radiation oncology Madison, WI, USA: Medical Physics Publishing; 1999. p1073.)

Radiation beam uniformity is one of a number of characteristics required for high energy x-ray beams to be useful in radiation therapy treatment. Verification of radiation beam uniformity in a plane perpendicular to the direction of the beam is particularly important for linear accelerators [1]. Uniformity must be maintained independent of the orientation or direction of the beam. Uniformity can be evaluated using various methods depending on the accelerator manufacturer's specifications. Typically the largest field size is evaluated at a particular depth in water and uniformity is specified over the central 80% of the beam. Figure 1 is an idealized two dimensional plot of radiation beam intensity across a typical accelerator photon beam. Beam uniformity is specified in terms of flatness and symmetry. Acceptable beam flatness and symmetry is produced by the combination of proper beam steering and a carefully designed filter. The accelerated electron beam is steered to a specific location (position) and exit orientation (angle) as it strikes the target (Figure 2). Beam steering is accomplished using two sets of steering coils. One set is located on the solenoid of the waveguide (Figure 2) and the second set is located in the electron beam transport section prior to the beam striking the target (not shown in Figure 2[2].

The accelerator system evaluates beam uniformity internally using the ion chambers located below the flattening filter. The beam current measured in each quadrant is compared and any asymmetry exceeding the specification results in a system interlock. An active system interlock takes the accelerator out of service until the problem is resolved. This interlock system prevents hazards that can cause serious injury to the patient or damage to the equipment. While the interlock system is extremely important, major interlocks disrupt workflow and retard clinical efficiency by forcing machine down time where patients can not be treated.

In this study we seek to characterize the process of medical linear accelerator beam steering and demonstrate, retrospectively, the ability to provide a level of control using the statistical process control (SPC) methodology [3-11]. For a generalized system, process performance data varies naturally. The information in the variation of a process is important for an understanding of how the process is performing. SPC is primarily a tool for understanding normal and abnormal variations [3,4]. SPC is used globally in manufacturing and business management to provide an ongoing evaluation of the stability and/or variability of a process [3,4]. Its success rests upon the fact that any process can be mapped by a series of inputs and outputs. Our ability to measure and effectively evaluate the variation of a critical subset of these inputs and outputs can provide an objective basis for timely intervention to maintain a high quality product - the desired beam flatness and symmetry for a medical linear accelerator. The work reported here test the hypothesis that SPC can ensure consistency of beam uniformity and indicate when intervention is required to correct non-uniformity prior to the actuation of a system interlock (thus saving valuable machine down time).

## II. Materials and Methods

Monitoring of radiation beam flatness and symmetry is critical to the quality of external beam radiation treatment delivery. It is possible to produce a beam with flatness that is within ± 3% of the central axis dose and has symmetry that does not exceed 2% across the central 80% of the defined field dimensions (Figure 1) [12]. Various manufacturers define flatness and symmetry differently. One common definition (Varian Medical Systems, Palo Alto, CA) of beam flatness is the maximum plus and minus variation from the mean beam intensity within the central 80% area of full width half maximum intensity. Likewise, linear symmetry is defined as the maximum variation of points symmetric to the central axis [13]. Modern linear accelerators utilize interlock systems to determine if predefined limits have been exceeded and will not allow the system to continue to operate once these limits are reached. The accelerator system used in this study, a 21 EX Varian C-series linac (Varian Medical Systems, Palo Alto, CA), performs a self-diagnostic test and creates Morning Checkout (MC) files during the initial warm-up at the start of each treatment day. Morning warm-up measurements are typically made at a single standard gantry angle and the accelerator feedback system is designed to maintain this setting as the gantry angle varies. The MC files are stored on the console computer but are not used for any specific analysis. Steering coil currents (amperes) for the transverse and radial plane position and angle steering coil currents are stored in the MC files.

The Varian 21EX at one of our regional affiliates experienced down time related to a water leak in the gantry stand. A fine crack in the water circulation tube dripped water on the electrical harness. This leak had persisted for months based on the amount of water found in the bottom of the stand and the fact that our records did not indicate any notable change in the water level. The leak was detected when there was an EXQT (total asymmetry exceeds 2% of symmetrical adjustment) interlock fault that would not clear. This interlock is an indication that the beam symmetry is outside specifications. Following repair, all beams were scanned and adjusted to ensure beam flatness, symmetry, and absolute dose outputs were within operating specifications.

Data from September 2009 (annual medical linear accelerator calibration) until two weeks following the beam steering failure in June 2010 were evaluated. The steering coil current (SCC) data were downloaded and manually extracted to Excel file format. The transverse and radial - positioning and angle SCC for photon beam energies were evaluated using average and range (Xbar-R) process behavior charts (Mintab v16, Minitab, Inc., State College, PA). The evaluation was limited to 6 MV and 15 MV photon beam data only.

### A. Process Control Charts (PCC)

To monitor a process, typically two control charts are created: 1) an average chart for subgroup averages, x, and 2) a range chart for subgroup ranges, R. The centerline for the subgroup average chart is *x*, which is the average of all the subgroup averages. The centerline for the range chart is *R*, which is the average of all the subgroup ranges. The daily recorded SCC (transverse angle, transverse position, radial angle, and radial position) values were regrouped into nominal weekly values (subgroup of size n = 5 days/week). Values from the first 20 weeks were used to calculate the limits (upper control limit (UCL) and lower control limit (LCL)). The average chart will have an upper threshold (A_u_), centerline (A_c_) and lower threshold (A_l_) [6,7] defined as

(1) $$A_{u}=x+6\frac{R}{d_{2}\sqrt{n}}=\text{UCL}$$

$$A_{c}=x=\left[{\left(\sum {\text{d}}_{\text{t}}\right)}_{\text{t}=1....\text{T}}\right]∕\text{T}$$

where T = 20 and

d_t _= mean SCC for treatment week number (t)

$$A_{l}=x-6\frac{R}{d_{2}\sqrt{n}}=\text{LCL}$$

where the factor '6' sets the number of standard deviations for the limit margins and n is equal to 5. Similarly, the range chart will have an upper threshold (R_u_), centerline (R_c_) and lower threshold (R_l_) [6,7] defined as

(2) $$R_{u}=\left(1+6\frac{d_{3}}{d_{2}}\right)R=\text{UCL}$$

$$R_{c}=R=\left[{\left(\sum {\text{R}}_{\text{t}}\right)}_{\text{t}=1.....\text{T}}\right]∕\text{T}\;\text{where}\;{\text{R}}_{\text{t}}=\left|{\text{d}}_{\text{tmax}}-{\text{d}}_{\text{tmin}}\right|$$

where T = 20 and

d_tmax _and d_tmin _= maximum and minimum SCC for

treatment week number (t)

$$R_{l}=\left(1-6\frac{d_{3}}{d_{2}}\right)R=\text{LCL}$$

The quantities d_2 _and d_3 _are correction factors that reflect the non-normality of the distribution of range values and also depend on the subgroup size n. The range limits have an asymmetric distribution about the mean range because the range is a positively skewed value and cannot be less than zero. It can be seen from equations (1) and (2) that only the quantities *x, R *and √n need to be computed to set action thresholds for the process control charts. The implication of exceeding the average or range chart limits on the process is thoroughly presented in SPC literature and an extensive review is beyond the scope of this work [3-7]. In general, data that exceeds the average chart limits suggest a special or non-random cause and/or an uncontrolled process, while exceeding the range chart limits indicates a process that is uncontrolled.

The control limits (UCL and LCL) were set using ± 6 standard deviations from the mean (equations 1 and 2). The chance of a random normal value exceeding the limits is extremely small. Typically limits are set using 3 standard deviations encompassing 99.7% of the normal distribution [4,6,8]. The cost of intervention (machine downtime, physics and engineering man-hours, scanning equipment, etc.) is considerable and a higher threshold limit appeared to be reasonable. Setting the limits at ± 6 standard deviations encompasses 99.9999998% of the normal distribution. The probability of a Type I error (a signal indicating an alarm incorrectly) is reduced and therefore the cost of unwarranted intervention is reduced as well. While there is an increase in the probability of a Type II error (a signal indicating there is no alarm when in fact a problem exist), this risk is balanced and offset by the existence of the interlock system. The data analysis was also performed using the 3 standard deviation control limits to gather a baseline understanding of how changing the limits may impact the investigation of or intervention in the process.

There are a number of standard tests other than the UCL and LCL that can be applied to PCC that assist in detecting a change in the process resulting from special causes or conditions [3,4],

a. *k *number of points in a row, on the same side of the center line

b. *k *number of points in a row, all increasing or decreasing

c. *k *number of points in a row, alternating up and down

d. *k *out of *k+1 *points,> 2 standard deviations from the center line on the same side

e. *k *out of *k+1 *points > 1 standard deviation from the center line on the same side

f. *k *number of points in a row within 1 standard deviation from the center line on either side

g. *k *number of points in a row > 1 standard deviation from the center line on either side

These can be considered as low-alarm or warning indicators. Low-alarm indicators represent a trend with a specific probability. Determining which low-alarm indicators to apply can be decided by using historical data to create a run chart. A run chart is simply a plot of individual or subgroup data points which allows the investigator to broadly characterize the process data. Reviewing the characteristics of the process data sampled can assist in determining which low alarm indicators may be most useful.

Two low-alarm indicators (a & d) were used in this study: 1) the observation of nine consecutive weekly samples on one side of the mean and 2) the observation of two out of three consecutive weekly samples greater than two standard deviations from the mean but not exceeding the UCL or LCL. For the first low-alarm indicator, the probability of a single value on one side of the mean but not exceeding the limit is 0.5. The probability of nine in a row is (0.5)^9 ^or ~ 0.002. The chance of this occurring is similar to a single value exceeding 3 standard deviations. For the second low-alarm indicator, the probability of a single value on one side of the mean being less than two standard deviations from the mean is 0.475. Also the probability of a single value on one side of the mean being greater than two standard deviations from the mean is 0.025. The probability for this low-alarm is then (0.475 × 0.025 × 0.025) or 0.0003. This alarm indicates a lack of process control and an acute shift in the mean. It is a powerful warning indicator whose probability of occurrence is smaller than that of a single value exceeding 3 standard deviations.

## III. Results and discussion

**Figure 3:**
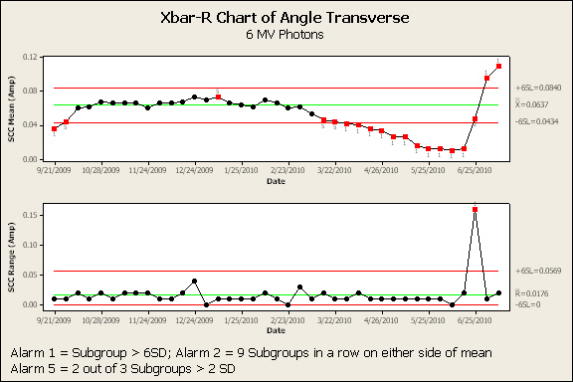
**Process control charts (average and range charts) for 6 MV photon beam transverse angle steering coil current.** Note: Red data points indicate an alarm has been signaled. Valid alarms occur after the first 20 data points which establish the calculated control limits.

**Figure 4:**
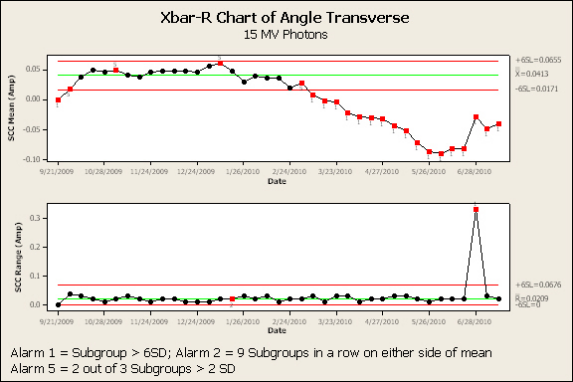
**Process control charts (average and range charts) for 15 MV photon beam transverse angle steering coil current**. Note: Red data points indicate an alarm has been signaled. Valid alarms occur after the first 20 data points which establish the calculated control limits.

**Figure 5:**
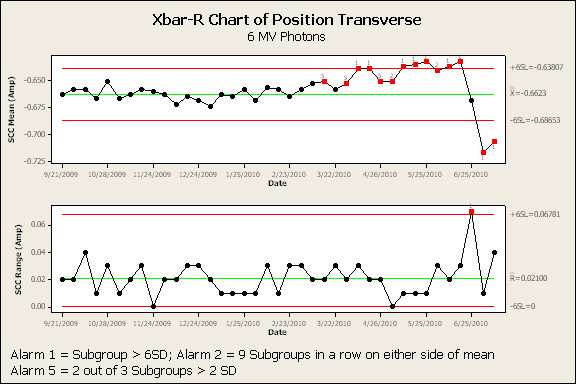
**Process control charts (average and range charts) for 6 MV photon beam transverse position steering coil current**. Note: Red data points indicate an alarm has been signaled. Valid alarms occur after the first 20 data points which establish the calculated control limits.

**Figure 6:**
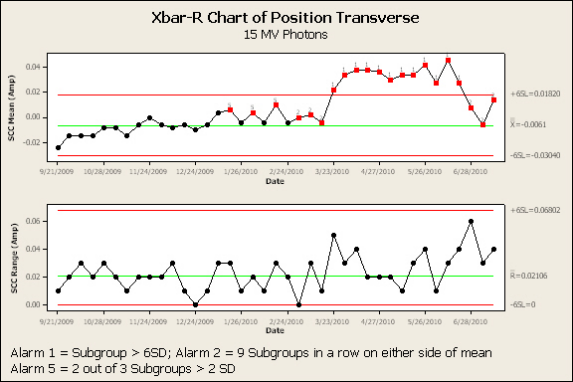
**Process control charts (average and range charts) for 15 MV photon beam transverse position steering coil current**. Note: Red data points indicate an alarm has been signaled. Valid alarms occur after the first 20 data points which establish the calculated control limits.

**Figure 7:**
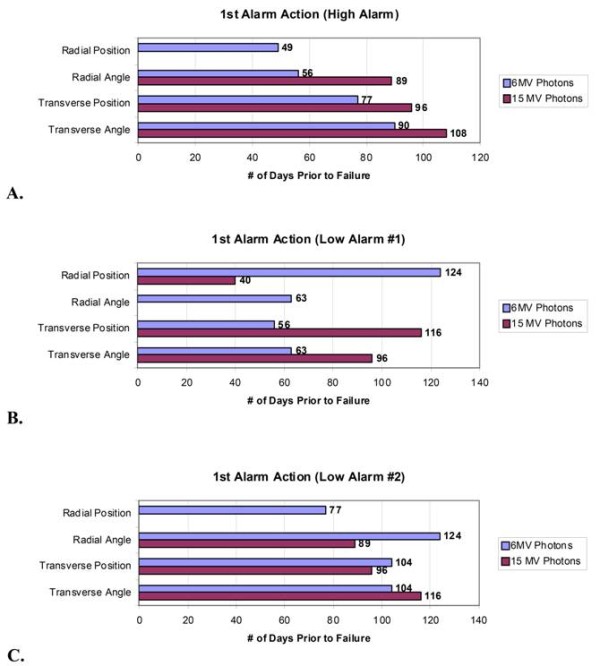
**Process control chart warning results**. Bars indicate the number of days prior to failure that an action level threshold was first triggered for each of the monitored steering coil parameters. A - High Alarm triggered indicating parameters exceeding ± 6 standard deviations of the mean weekly sample value. B - Low alarm indicating the observation of nine consecutive weekly samples on one side of the mean. C - Low alarm indicating the observation of two out of three consecutive weekly samples greater than two standard deviations from the mean but not exceeding the high alarm limit.

Figure 3 and 4 are the SPC results of the transverse angle SCC for the 6 MV and 15 MV beams, respectively. The high-alarm action limit is indicated by a 1 below a red data point. The low-alarms are indicated by a 2 [nine weekly samples on one side of the mean] or a 5 [two out of three consecutive weekly samples greater than two standard deviations from the mean] below a red data point respectively. SPC analysis of transverse angle SCC for 6 MV and 15 MV indicated a high-alarm on April 14^th ^and March 13^th ^- 90 and 108 days prior to failure, respectively. In each case, a downward trend in this parameter continued, with high-alarm, until failure. Transverse position (Figure 5 and 6) and radial angle SCC for both energies indicated a low-alarm starting in March or April. Transverse position SCC results show sensitivity similar to the transverse angle results. Analysis of the SCC in the radial plane did not indicate the same sensitivity as early as the SCC in the transverse plane. All the results are summarized in Figure 7 and show the number of days prior to failure that an alarm was triggered.

Comparison of PCC using control limits of 3 standard deviations versus 6 standard deviations primarily indicates:

a. Low alarms are changed to high alarms requiring intervention.

b. There is an increase in the number of Type I errors observed.

The result of the comparison supports the use of 6 standard deviation control limits for monitoring the beam steering process.

Subsequent to the repair, continued monitoring using SPC requires the calculation of new limits based on the newly established operating SCC. The first twenty subgroups would be used to determine new limits and SPC then continues to monitor and evaluate process variation.

These results indicate that evaluation of beam steering currents using SPC would have provided an early indication of deviations in the current values prior to the actuation of the interlock and required unscheduled downtime. Results of the 6 MV transverse angle SCC results show the first low-alarm (5 - two out of three points greater than two standard deviations) on March 15, which was 104 days prior to failure with a high-alarm two weeks later. The low-alarm was fifteen weeks prior to actuation of the system interlock. The 15 MV transverse angle SCC show the first low-alarm (5- two out of three points greater than two standard deviations) on March 3, which was 116 days prior to failure, followed by a high-alarm one week later. These alarms confirm the diagnosis that the water leak and resulting damage persisted for a relatively long period of time. Investigation of this alarm would have initiated sixteen weeks prior to the actuation of the system interlock. The radial angle and radial position low-alarms gave the earliest warnings (124 days). In addition, the low-alarm indicators were effective in signaling a change in the established operating SCC of both energies. Overall, the low-alarm indicating two out of three points greater than two standard deviations was most effective in predicting a "permanent" shift in the mean SCC values. Further study of additional low-alarm indicators may establish which alarms are most predictive of a clinically significant change in beam steering.

Typically, standard control limits are calculated using three standard deviations to balance the cost-benefit as well as the Type I and II error probability. The use of six standard deviations for calculating the control chart limits was particularly economical in our study. The physics and engineering effort required to investigate a Type I error is significant. We believe a high threshold for action is desirable compared to current cost of unwarranted intervention or investigation.

The use of digital control systems in medical linear accelerators make them ideal for the incorporation of a SPC subsystem that can use the performance data generated to monitor consistency of operation. Quality of patient treatment delivery can be improved by ensuring consistent operating parameters relative to the baseline values determined at the time of accelerator commissioning [14]. The use of an SPC subsystem is non-invasive and simply samples data already generated by the accelerator. While SPC could be used to alert the physicist and service engineer to a change in operating parameters, any use as an active control system would need to have FDA clearance provided by the manufacturer.

## IV. Conclusion

The results of this study suggest that the use of the investigated SPC methodology with a six standard deviation high-alarm action limit would have prompted an investigation of the beam steering process prior to a significant change in beam flatness and symmetry and made detection of the water leak responsible for changes in SCC performance more likely. The impact of machine downtime on clinical operational efficiency would be minimized by the scheduling of planned maintenance to be performed without interruption of the clinic treatment schedule.

System interlocks provide an important safety function to safeguard the equipment from catastrophic failure and prevent unsafe clinical use when operating specifications are exceeded. SPC could be used to detect non-standard functioning of beam flatness and symmetry prior to these measurements creating beam interrupts and altering treatment times and schedules. This early warning alert could ultimately improve treatment delivery and clinical operational efficiency. The use of SPC to evaluate the operating parameters of critical accelerator systems has the potential to improve the quality of accelerator maintenance, treatment delivery and ultimately patient safety. SPC could be expanded to help provide a "smart-accelerator" system that communicates non-standard functioning of systems directly to the local service representative and physicist if automated.

## Competing interests

This project was partially supported by a grant from Varian Medical Systems, Inc.

## Authors' contributions

CMA designed the study, performed the data analysis and is the lead editor of the manuscript. CJH assisted in study design, data analysis as well as insight into the application of statistical process control methods. AHB performed the data capture and processing prior to the application of statistical process control and editing of the manuscript. MTM provided institutional support and played a pivotal role in manuscript editing for intellectual content. All the authors have read and approved the final manuscript.

## References

[B1] NCRP Dosimetry of x-ray and gamma-ray beams for radiation therapy in the energy range 10 kev to 50 Mev 1981 109 Report No. 69. Bethesda, MD: National Council on Radiation Protection and Measurements

[B2] Van Dyk J The modern technology of radiation oncology 1999 Madison, WI, USA: Medical Physics Publishing 1073

[B3] Stapenhurst T Mastering statistical process control 2005 Jordan Hill, Oxford, UK: Butterworth-Heinemann

[B4] Oakland JS Statistical process control 2008 Jordan Hill, Oxford, UK: Butterworth-Heinemann

[B5] Burr IW The effect of non-normality on constants for Xbar and R Charts Industrial Quality Control 1996 565 569

[B6] Manual on quality control of materials 1951 American Society for Testing and Materials;Philadelphia, PA, USA

[B7] Wheeler DJ Normality and the process control chart 2000 Knoxville. TN, USA: SPC Press

[B8] Neubauer DV Manual on Presentation of data and control chart analysis 2010 West Conshohocken, PA, USA: ASTM International

[B9] Wheeler DJ Chambers DS Understanding statistical process control 1992 Knoxville, TN, USA: SPC Press

[B10] Able C Bright M Quality control of external beam treatment delivery: mechanical parameters [abstract] Med Phys 2009 36 2428

[B11] Able CM Bright M Frizzell B Quality Control of High-Dose-Rate Brachytherapy: Treatment Delivery Analysis Using Statistical Process Control [abstract] Brachytherapy 2010 9 S64 S65 10.1016/j.ijrobp.2012.05.016 22749631

[B12] Kahn F The Physics of Radiation Therapy 2003 Philadelphia, PA, USA: Lippincott Williams & Wilkins

[B13] Varian Medical Systems 2006 High Energy Clinac Customer Acceptance Procedure, Revision E. Milpitas, CA, USA

[B14] Klein E Hanley J Bayouth J Yin F Simon W Dresser S Serago C Aguirre F Ma L Arjomandy B Liu C Sandin C Holmes T Task Group 142: Quality assurance of medical accelerators Med Phys 36 9 4197 4212 10.1118/1.3190392 19810494

